# Cleft Palate Induced by Mycophenolate Mofetil Is Associated with *miR-4680-3p* and *let-7c-5p* in Human Palate Cells

**DOI:** 10.3390/ncrna11010012

**Published:** 2025-02-06

**Authors:** Hiroki Yoshioka, Hanane Horita, Yosuke Tsukiboshi, Hisaka Kurita, Aya Ogata, Kenichi Ogata

**Affiliations:** 1 Faculty of Pharmacy, Gifu University of Medical Science, 4-3-3 Nijigaoka, Kani, Gifu 509-0293, Japan; 2 Department of Hygiene, Kitasato University School of Medicine, 1-15-1 Kitasato, Minami-ku, Sagamihara, Kanagawa 252-0374, Japan; 3 Laboratory of Medical Therapeutics and Molecular Therapeutics, Gifu Pharmaceutical University, 1-25-4 Daigaku-nishi, Gifu, Gifu 501-1196, Japan; 4 Section of Oral and Maxillofacial Oncology, Division of Maxillofacial Diagnostic and Surgical Sciences, Faculty of Dental Science, Kyushu University, 3-1-1 Maidashi, Higashi-ku, Fukuoka 812-8582, Japan

**Keywords:** mycophenolate mofetil, cleft palate, microRNA, cell cycle

## Abstract

**Background/Objectives**: Cleft palate is a birth defect associated with environmental and genetic factors. Disturbance of microRNAs (miRNAs) and exposure to medicinal agents during pregnancy can cause cleft palate. Although an association between medicine-induced cleft palate and miRNAs has been suggested, it remains to be fully elucidated. This study aimed to clarify the molecular mechanism underlying mycophenolate mofetil (MPM)-induced inhibition of cell proliferation and miRNA expression in human embryonic palatal mesenchymal (HEPM) cells. **Methods**: Cell viability, apoptosis, and cell cycle-related markers were evaluated 48 h after MPM treatment. In addition, miRNA levels and expression of their downstream genes were measured, and a rescue experiment was performed using *miR-4680-3p* and/or *let-7c-5p* inhibitors. **Results**: MPM dose-dependently reduced HEPM cell viability. Additionally, MPM treatment suppressed cyclin-D1, cyclin E1, cyclin-dependent kinase (CDK)-2, and CDK6 expression in HEPM cells. Furthermore, MPM upregulated *miR-4680-3p* and *let-7c-5p* expression and downregulated the downstream genes of each miRNA. Moreover, *miR-4680-3p* and/or *let-7c-5p* inhibitors alleviated MPM-induced inhibition of cell proliferation. **Conclusions**: These results suggest that MPM-induced cleft palate is associated with *miR-4680-3p* and *let-7c-5p* expression in HEPM cells.

## 1. Introduction

Cleft palate (CP) is one of the most common birth defects worldwide, affecting approximately 1 in every 500 babies in Asia. It was reported that 70% of cleft lip (CL) with or without CP (CL/P) are non-syndromic, while the others are syndromic [1]. In humans, the palate forms in two stages, called primary and secondary palates. Secondary palate formation happens approximately between the 6th and 10th week of gestation through mesenchymal cell proliferation and epithelial cell fusion, wherein the two pieces of tissue, called palatal shelves, grow downward on either side of the tongue around the 6th–7th week. Then, around the 7th–8th week, the tongue is retracted between the palatal shelves, which then elevate and fuse above the tongue and primary palate. Finally, the medial epithelial seam breaks down through either apoptosis or migration toward the epithelial triangles on both the oral and nasal sides or by epithelial–mesenchymal transition until the 10th week. Any delay or alteration in apoptosis and/or cell cycle arrest during 6–10 weeks of gestation can result in CP [1].

The etiology of CP is associated with both genetic and environmental factors [2,3]. Many genes are known to affect the proliferation of palatal shelves or bind each palatal shelf. Li et al. reported 131 genes involved in CP obtained from a systematic review [4]. For example, p63 is a key mediator of epidermal development, and deletion of p63 in mice leads to CP, especially in the squamous epithelia and epidermis [5]. Meanwhile, E-cadherin plays a crucial role in epithelial cell–cell adhesion [6]. The mutation of E-cadherin was associated with CLP through the downregulation of β-catenin signaling [7,8]. Transforming growth factor beta (TGFβ) signaling is one of the major signaling cascades for craniofacial development [9], and TGFβ2 knockout in epithelial cells induced CP in mice [10]. Furthermore, Sprouty2 regulates mesenchymal cell proliferation, and its deletion induced CP through the downregulation of fibroblast growth factor signaling [11]. Environmental factors such as medicines, alcohol, and smoking, are suggested to increase the risk of CP through the inhibition of crucial genes or signaling pathways [12]. Additionally, a recent investigation demonstrated that other environmental factors such as exposure to particulate matter 2.5 microns or less in diameter (PM2.5) are involved in CP development [13,14]. However, the mechanisms that induce CP are not fully understood.

MicroRNAs (miRNAs) are single-strand non-coding RNAs (20–25 nucleotides). Since the miRNA lin-4 from *Caenorhabditis elegans* was first discovered in 1993, miRNA processing and functional machinery have been reported, and thousands of miRNAs have been identified across the species [15,16]. The majority of miRNAs are processed through the canonical miRNA pathway employing the Drosha/DGCR, Dicer/TRBP, and Exportin-5/RAN-GTP complexes [17,18]. miRNAs negatively modulate gene expression by binding to 3’-untranslated regions, resulting in the inhibition of the protein translation or degradation of mRNA transcripts [19,20]. miRNAs play a vital effect in the formation of palatal shelves [21,22]. miRNAs are associated with mesenchymal palatal cell proliferation by modulating its downstream genes [23,24]. Moreover, our group and other researchers have reported that medicines such as *all-trans* retinoic acid (*at*RA) and dexamethasone modulated specific miRNAs and their downstream genes in human embryonic palatal mesenchymal (HEPM) cells and other related cell types [24,25,26]. For example, Yoshioka et al. demonstrated that dexamethasone suppressed mouse embryonic palatal mesenchymal cell viability through *mmu-miR-130a-3p* reduction and upregulation of *Slc24a2* expression levels [24]. Yoshioka et al. also showed that *all-trans* retinoic acid inhibited HEPM cell viability by the induction of *hsa-miR-4680-3p* and the downregulation of *ERBB2* and *JADE1* expression levels [26]. In addition, *at*RA administration in pregnant mice upregulated *mmu-miR106a-5p* in the palatal shelves by reducing *Tgfb2* expression levels [25]. Although studies focusing on miRNAs and medicine-induced CP are being gradually published, their availability is still limited.

Mycophenolate mofetil (MPM) is a mycophenolic acid prodrug that has been approved as an immunosuppressive agent. MPM has been prescribed as an alternative medicine for many autoimmune diseases such as nephrotic syndrome and lupus nephritis [27]. Although MPM shows potent immunosuppressive efficacy and fewer side effects than previously developed anti-proliferative immunosuppressive agents [28], several studies have reported that maternal exposure to MPM causes embryo teratogenicity, including CL/P and microtia [29,30]. Using the GeneCard database, Lin et al. reported that several molecules (MDM2, RPL5, and TP53) may be involved in MPM-induced CL/P [31]. Since mycophenolic acid upregulated *miR-142* and *miR-146* in lupus CD4-positive T cells [32], miRNAs should be a target for mycophenolic acid-induced pharmacological/toxicological effects. However, the involvement of miRNA regulation in MPM-induced CP remains unknown. In the present study, we investigated the involvement of miRNAs in MPM-induced inhibition of cell proliferation using HEPM cells.

## 2. Results

### 2.1. MPM Inhibits Cell Proliferation via G1 Arrest in HEPM Cells

**Figure 1:**
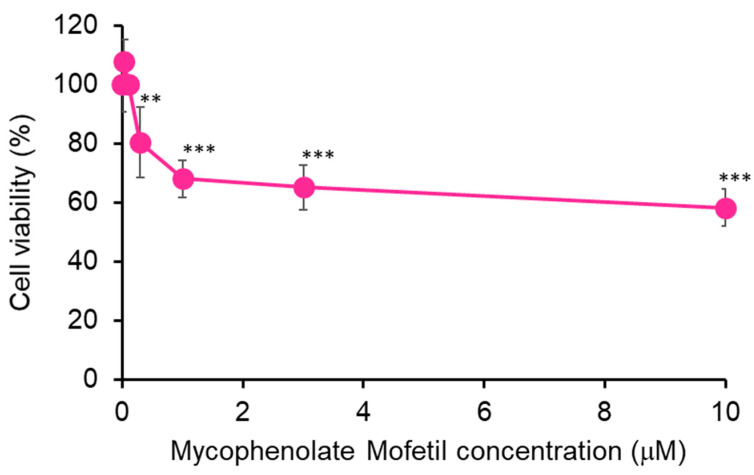
Inhibition of cell proliferation by MPM in HEPM cells. Proliferation of HEPM cells treated with MPM (0.01, 0.03, 0.1, 0.3, 1, 3, and 10 μM) for 48 h. Data are presented as the mean ± standard deviation (SD). ** *p* < 0.01, and *** *p* < 0.001 versus control (n = 6).

First, we performed a cell viability assay to determine whether MPM reduced cell viability in HEPM cells. MPM treatment inhibited the viability of HEPM cells in a dose-dependent manner (Figure 1). We selected 1 μM MPM for the following experiments since the suppression effect plateaued at this dose.

**Figure 2:**
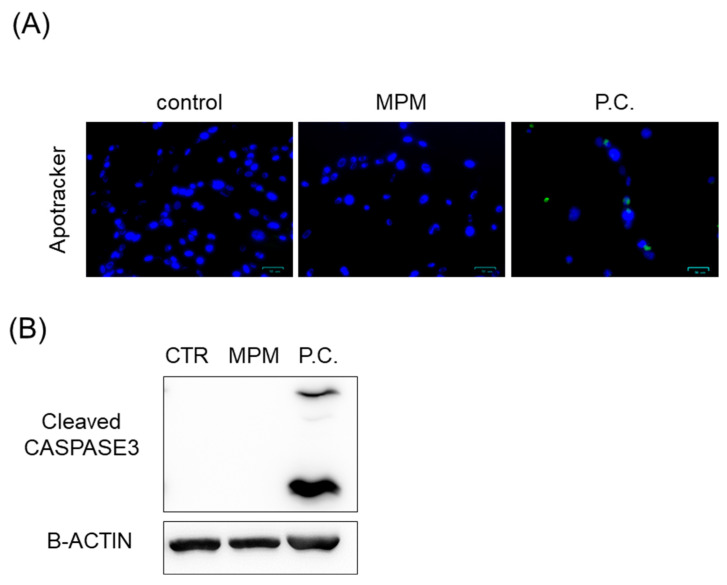
MPM-induced cell proliferation is not associated with apoptosis in HEPM cells. (**A**) Apotracker staining of HEPM cells after treatment with 1 μM MPM for 48 h. The nuclei were counterstained with Hoechst 33342. Copper dichloride (500 μM 24 h) was used as a positive control. Scale bar, 50 μm. (**B**) Western blotting of HEPM cells treated with 1 μM MPM for 48 h. β-ACTIN was used as an internal control. Copper dichloride (500 μM 24 h) was used as positive control (P.C.).

Medicine-induced inhibition of mesenchymal palatal cell proliferation induces apoptosis and cell cycle arrest [33,34,35]. Therefore, we tested the effect of apoptosis and cell cycle arrest in MPM-treated HEPM cells. As shown in Figure 2A, treatment with MPM did not increase apoptosis-positive cells as monitored by Apotracher, whereas copper dichloride (positive control) treatment activated apoptosis-positive cells. To support the results of Apotracker, we demonstrated that the protein expression of cleaved caspase-3, an indicator of apoptosis, was not increased by MPM treatment (Figure 2B).

**Figure 3:**
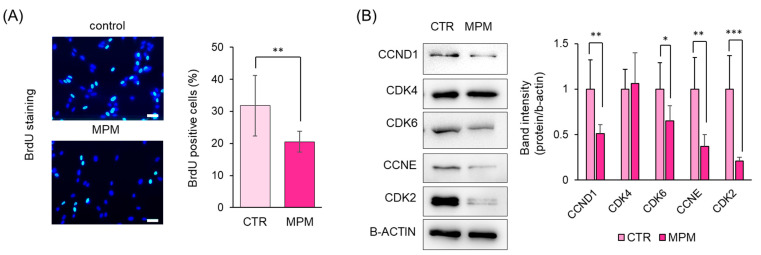
MPM-induced cell proliferation is associated with G1 arrest in HEPM cells. (**A**) BrdU staining (green) of HEPM cells after treatment with 1 μM MPM for 48 h. The nuclei were counterstained with Hoechst 33342 (blue). Scale bar, 50 μm. The graph shows the quantification of BrdU-positive cells. Data are presented as the mean ± standard deviation (SD). ** *p* < 0.01 (n = 6). (**B**) Western blotting of HEPM cells treated with 1 μM MPM for 48 h. β-ACTIN was used as an internal control. * *p* < 0.05, ** *p* < 0.01, *** *p* < 0.001 (n = 3).

Next, we monitored the cell cycle progression using a BrdU incorporation assay and found that the number of BrdU-positive cells was significantly reduced by MPM treatment (Figure 3A). To further investigate the molecular mechanisms underlying MPM-induced cell cycle arrest (G1-arrest), we tested cyclins and cyclin-dependent kinases (CDK) and found that MPM treatment reduced CCND1, CCNE, CDK2, and CDK6 levels, whereas those of CDK4 were comparable (Figure 3B). These results indicate that MPM induces cell cycle arrest (G1-arrest) by suppressing CCND1/CDK6 and CCNE/CDK2 in HEPM cells.

### 2.2. MPM Modulates let-7c-5p/miR-4680-3p and Its Downstream Genes in HEPM Cells

**Figure 4:**
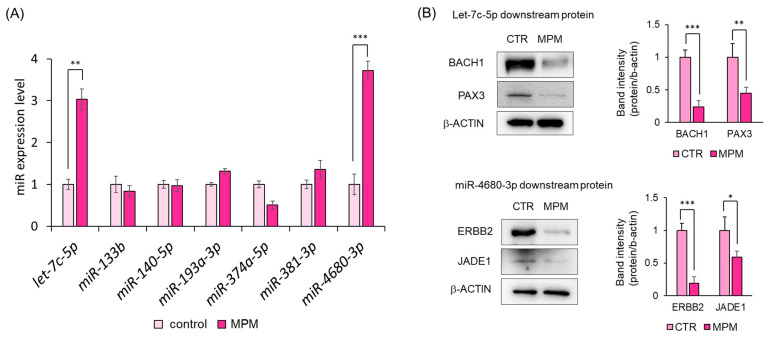
MPM upregulated *let-7c-5p* and *miR-4680-3p* in HEPM cells. (**A**) Quantitative RT-PCR of *let-7c-5p*, *miR-133b*, *miR-140-5p*, *miR-193a-3p*, *miR-374a-5p*, *miR-381-3p*, and *miR-4680-3p* after treatment of HEPM cells with 1 μM MPM for 48 h. Data are presented as the mean ± standard deviation (SD). ** *p* < 0.01 and *** *p* < 0.001 (n = 3). (**B**) Western blotting of HEPM cells treated with 1 μM MPM for 48 h. β-ACTIN was used as an internal control. * *p* < 0.05, ** *p* < 0.01, *** *p* < 0.001 (n = 3).

Recently, miRNAs have been suggested to be associated with the CL/P etiology [21,22]. Suzuki and Li et al. identified miRNAs associated with human CP-related genes by performing systematic reviews, bioinformatic reviews, cell proliferation assays, and qPCR [4,36]. Moreover, Fu et al. identified two miRNAs from a CL/P patient database and in vitro experiments [23]. In the present study, we measured the expression of seven miRNAs (*let-7c-5p*, *miR-133b*, *miR-140-5p*, *miR-193a-3p*, *miR-374a-5p*, *miR-381-3p*, and *miR-4680-3p*) using qPCR, as induction of these seven miRNAs reduced HEPM cell viability [4,23,37]. We found that MPM treatment significantly increased *let-7c-5p* and *miR-4680-3p* expression, whereas the expression of the other five miRNAs was unaltered in HEPM cells (Figure 4A). To further investigate the effects of *let-7c-5p* and *miR-4680-3p* on downstream genes (*BACH1* and *PAX3* for *let-7c-5p* and *ERBB2* and *JADE1* for *miR-4680-3p*) [26,38], we conducted a Western blot analysis and found that MPM treatment suppressed BACH1, PAX3, ERBB2, and JADE levels (Figure 4B). These findings indicate that MPM modulates *let-7c-5p* and *miR-4680-3p* upregulation and inhibition of their downstream genes.

### 2.3. Inhibition of let-7c-5p and/or miR-4680-3p Alleviated MPM-Induced Cell Proliferation Activity in *HEPM* Cells

**Figure 5:**
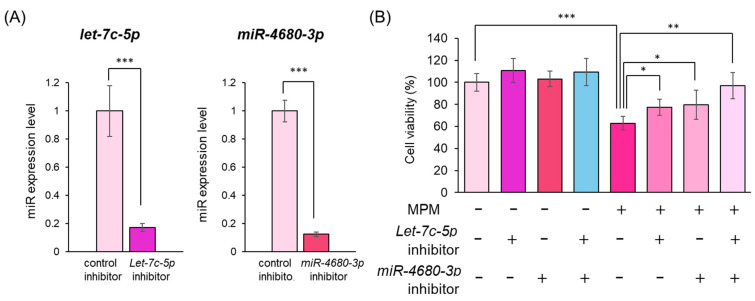
Inhibition of *let-7c-5p* and *miR-4680-3p* alleviated MPM-induced cell proliferation inhibition in HEPM cells. (**A**) Quantitative RT-PCR analysis of *let-7c-5p* or *miR-4680-3p* expression after transfecting HEPM cells with *let-7c-5p* inhibitor or *miR-4680-3p* inhibitor for 24 h. Data are presented as the mean ± standard deviation (SD). *** *p* < 0.001 (n = 3). (**B**) Proliferation of HEPM cells treated with 1 μM MPM and/or *let-7c-5p* inhibitor and/or *miR-4680-3p* inhibitor for 48 h. Data are presented as the mean ± standard deviation (SD). * *p* < 0.05, ** *p* < 0.01, and *** *p* < 0.001 (n = 6).

To further investigate the contributions of *let-7c-5p* and *miR-4680-3p* inhibition, we transfected HEPM cells with *let-7c-5p* and/or *miR-4680-3p* inhibitors to examine whether *let-7c-5p* and/or *miR-4680-3p* alleviated the inhibition of HEPM cell proliferation following MPM treatment. Transfection with *let-7c-5p* and *miR-4680-3p* inhibitors suppressed the expression of their respective targets by more than 80% under our experimental conditions (Figure 5A). Finally, we investigated whether treatment with *let-7c-5p* and/or *miR-4680-3p* inhibitor rescued MPM-induced cell proliferation reduction or not. We found that *let-7c-5p* or *miR-4680-3p* inhibition partially alleviated the MPM-induced reduction in cell proliferation (Figure 5B). Moreover, treatment with both *let-7c-5p* and *miR-4680-3p* inhibitors fully protected MPM-induced cell proliferation inhibition in HEPM cells (Figure 5B). These results suggest that *let-7c-5p* and *miR-4680-3p* were associated with MPM-induced inhibition of HEPM cell proliferation.

## 3. Discussion

In this study, we examined the role of miRNAs in MPM-induced toxicity in HEPM cells. MPM treatment reduced the cell viability in a dose-dependent manner (Figure 1) and downregulated the expression of CCND1, CCNE, CDK2, and CDK6 in HEPM cells (Figure 3B). Several reports have suggested that MPM-induced pharmacological effects derive from purine synthesis (inosine-5’-monophosphate dehydrogenase) inhibition [39,40]; therefore, MPM may induces cell cycle arrest in HEPM cells (Figure 3A,B). Additionally, MPM treatment induced *let-7c-5p* and *miR-4680-3p* expression and downregulated the expression of their downstream genes (Figure 4B). Notably, the inhibition of *let-7c-5p* or *miR-4680-3p* partially alleviated MPM-induced cell inhibition, and inhibition of both *let-7c-5p* and *miR-4680-3p* fully protected HEPM cells against MPM-induced cell proliferation inhibition (Figure 5B).

Cyclins and CDKs play crucial roles in the regulation of cell cycle events [41]. When cells in the G0 phase enter the cell cycle, CDK4/CDK6 forms active complexes with CCND and other proteins, such as phosphorylated retinoblastoma protein (pRb), which activates the transition step from the G1 to S phase [42]. Moreover, the CDK2 and CCNE complex phosphorylates and inactivates Rb family members [43]. The subsequent release of transcription factors (e.g., E2F) allows the cells to transition from G1 to S phase. Many cancers are known to increase CCND levels, and CCND activates signaling pathways, such as the MAPK kinase and PI3K/Akt pathways [44]. CCNE overexpression accelerates G1 phase progression in cancer patients [45]. CDK2 inhibition reduces the viability of human colon cancer cells [46]. CDK4/6 inhibitors, such as palbociclib, ribociclib, and abemaciclib, have been approved for patients with HER2-negative breast cancer [47]. We demonstrated that the number of BrdU-positive cells was significantly reduced using MPM treatment (Figure 3A). Since the BrdU incorporation assay is an indicator of S phase, we investigated the molecular mechanism related to G1 phase proteins and found that MPM reduced CCND1, CDK6, CCNE, and CDK2 levels (Figure 3B). Therefore, MPM-induced cell viability reduction consists of G1-arrest through the suppression of CCND1/CDK6 and CCNE/CDK2 in HEPM cells. However, as our experiments (BrdU assay and Western blotting) have some limitations, we need to use alternative methods such as FACS analysis and fluorescently labeled assay in the future [48,49].

Several studies have reported that miRNA-gene networks are involved in craniofacial development [50,51]. Li et al. showed that overexpression and/or knockout of *mmu-miR-17-92* cluster in mice leads to CL and CP by modulating the BMP signaling pathway [52]. Polymorphisms in miR-140 and miR-4260 are associated with non-syndromic CL/P in humans [53,54]. Li and Suzuki et al. showed that several miRNAs (*hsa-miR-133b*, *hsa-miR-140-5p*, *hsa-miR-374a-5p*, *hsa-miR-381a-3p*, and *hsa-miR-4680-3p*) are involved in the development of human palate using a combination of multiple experiments such as systematic reviews, bioinformatics analyses, and cell viability assays [4,36]. Fu et al. reported that *hsa-let-7c-5p* and *hsa-miR-193a-3p* are associated with CP using an in vitro experiment with HEPM cells [23]. To test the involvement of the above miRNAs, we measured the seven (*hsa-let-7c-5p*, *hsa-miR-133b*, *hsa-miR-140-5p*, *hsa-miR-193a-3p*, *hsa-miR-374a-5p*, *hsa-miR-381a-3p*, and *hsa-miR-4680-3p*) miRNAs and demonstrated that MPM significantly induced *let-7c-5p* and *miR-4680-3p* expression levels in HEPM cells (Figure 4A). In addition, *let-7c-5p* or *miR-4680-3p* specific inhibitors partially alleviated, and in combined treatment, fully protected, against MPM-induced suppression of HEPM cell proliferation (Figure 5B), indicating that *let-7c-5p* and *miR-4680-3p* play an important role in MPM-induced toxicity. Human *let-7c-5p* is located on chromosome 21 and is involved in cell proliferation [55]. Overexpression of *let-7c-5p* suppresses human breast cancer and osteoblasts by downregulating CCDN1 [56,57]. Moreover, we previously reported that *let-7c-5p* and its inhibitor partially attenuated the phenobarbital-induced cell viability reduction by modulating BACH1 and PAX3 in HEPM cells [38]. Human *miR-4680-3p* is located on chromosome 10 and is expressed in gastric cancer [58]. *miR-4680-3p* and *miR-4680-3p* inhibitors partially attenuated *all-trans* retinoic acid- and phenytoin-induced inhibition of cell proliferation through the regulation of ERBB2 and JADE1 [26,59]. Since these miRNAs are associated with several genes, they may play an important role in palate development by modulating downstream genes.

miRNAs negatively regulate downstream genes [60]. We previously identified that *let-7c-5p* inhibitor induces two CP-associated genes (*BACH1* and *PAX3*) in humans [38]. As expected, both genes were downregulated following MPM treatment (Figure 4B). *BACH1* is ubiquitously expressed in mammals and is involved in multiple events, such as cell cycle and proliferation, through the modulation of the Wnt/β-catenin signaling pathway [61,62]. *PAX3* is essential for neural crest development. Knockout of *PAX3* results in CP via downregulation of the BMP signaling pathway [63], and a *PAX3* variant is associated with non-syndromic CL/P in humans [64]. As for *miR-4680-3p*, *ERBB2* and *JADE1* have been reported as downstream genes in HEPM cells [26]. We found that the expression of both genes was attenuated by MPM treatment in HEPM cells (Figure 4B). ERBB2 (HER2) is a member of the ERBB-receptor tyrosine kinase family, which includes the epidermal growth factor receptor (EGFR). ERBB2 is a target in ERBB2-positive breast cancer [65]. ERBB2 is associated with cell proliferation, migration, and differentiation through the modulation of signaling cascades such as the MAPK/ERK and PI3K/AKT/mTOR pathways [66]. ERBB2 downregulation by *all-trans* retinoic acid or siRNA reduced cell viability via the ERK1/2 signaling pathway in HEPM cells [26]. JADE1 (PHF17) is a transcription factor and its inhibition reduces cell viability in cultured epithelial cell lines and primary fibroblasts [67]. JADE1 is known to regulate the Wnt/β-catenin signaling pathway [68]. JADE1 inhibition by siRNA reduces HEPM cell viability [26]. As these genes are associated with several signaling pathways related to proliferation, these miRNA–mRNA networks may play a crucial role in palate development by modulating these signaling pathways.

## 4. Materials and Methods

### 4.1. Cell Culture

HEPM cells were purchased from American Type Culture Collection (CRL-1486, Manassas, VA, USA) and kept in Minimum Essential Medium Eagle-alpha modification medium (αMEM; Fujifilm-Wako Pure Chemical Corporation, Osaka, Japan) supplemented with 10% fetal bovine serum (Millipore-Sigma, St Louis, MO, USA), penicillin (10 U/mL), and streptomycin (10 μg/mL; Fujifilm-Wako Pure Chemical Corporation). The cells were kept at 37 °C in a humidified atmosphere containing 5% CO_2_.

### 4.2. Cell Viability Assay

HEPM cells were seeded at 5000 cells per 96-well plate (n = 6) and treated with several concentrations (0–10 μM) of MPM (Tokyo Kasei Co. Ltd., Tokyo, Japan) after 24 h of cell seeding. After 48 h of treatment, the cell viability was measured using Alamar Blue (Bio-Rad Laboratories, Hercules, CA, USA).

### 4.3. Apoptosis Assay

HEPM cells were seeded at 10,000 cells per 8-well chamber slide (Biomedical Sciences Inc., Tokyo, Japan) and treated with 1 μM MPM or vehicle after 24 h of seeding. After 48 h of treatment, apoptosis-positive cells were calculated using ApoTracker Green (BioLegend, San Diego, CA, USA) according to our previous study [59,69]. Copper dichloride was used as a positive control and Hoechst 33342 (Nacalai Tesque, Kyoto, Japan) was used as a nuclear counterstaining.

### 4.4. Western Blotting

HEPM cells were seeded at 200,000 cells per 35 mm dish and treated with 1 μM MPM or vehicle after 24 h of seeding. After 48 h of treatment, they were washed with PBS twice. To collect the protein, we added 100 μL ice-cold RIPA buffer (Nacalai Tesque) containing a protease inhibitor (Nacalai Tesque) and kept it on ice. After 5 min, we scraped the cells and sonicated the mixture (15%, 5 s, 3 times, Branson, Danbury, CR, USA). We subsequently centrifuged it (20,000× *g* for 20 min at 4 °C) and collected the supernatant as protein. The protein concentration was calculated as previously described [70,71]. Protein samples (10 μg) were applied to gradient (5–20%) precast sodium dodecyl sulfate-polyacrylamide gel (ATTO, Tokyo, Japan) and transferred onto polyvinylidene difluoride (PVDF) membranes using Trans Turbo Blot (Bio-Rad Laboratories). The antibodies used are listed in Table 1. Immunoreactive bands were visualized using the Western Blot Hyper HRP Substrate (Takara Bio, Shiga, Japan) and LuminoGraph II (ATTO). Band intensity was measured using Image J 1.54 software (NIH, Bethesda, MD, USA) [72].

### 4.5. Bromodeoxyuridine (BrdU) Incorporation Assay

HEPM cells were seeded at 10,000 cells per 8-well chamber slide (Biomedical Sciences Inc.) and treated with 1 μM MPM or vehicle (0.1% DMSO). After 48 h of treatment, the cells were incubated with BrdU (100 μg/mL, Sigma-Millipore, Burlington, MA, USA) as previously described [59,69].

### 4.6. Quantitative RT-PCR

HEPM cells were seeded at 200,000 cells per 35 mm dish and treated with 1 μM MPM or vehicle after 24 h of seeding. After 48 h of treatment, they were washed with PBS twice. To collect the total RNA, we extracted RNA from HEPM cells using a QIAshredder and miRNeasy Mini Kit (QIAGEN, Valencia, CA, USA) (n = 3–5) [73]. miRNA expression was evaluated according to our previous study [59]. The expression level of target miRNAs was standardized to U6 expression levels.

### 4.7. Rescue Experiments Using an miRNA Inhibitor

To rescue the effect of MPM, cells were treated with an miRNA inhibitor. We obtained miRNAs from BIONEER Ltd. (Daejeon, Republic of Korea) or Integrated DNA Technologies (IDT). HEPM cells were seeded at 5000 cells per 96-well plate (n = 6). After 6 h of cell seeding, the HEPM cells were treated with *hsa-7c-5p* inhibitor (3 pmol; BIONEER Ltd.), control miR inhibitor (3 pmol; BIONEER Ltd.), *hsa-iR-4680-3p* inhibitor (3 pmol: IDT, Coralville, IA, USA), or control miR inhibitor (3 pmol: IDT) using FuGENE SI Transfection Reagent (Promega, Madison, WI, USA), according to the manufacturer’s protocol. Cells were treated with 1 μM MPM 24 h after transfection. After 48 h of treatment, cell viability was evaluated using Alamar Blue.

### 4.8. Statistical Analysis

Comparisons between two or more groups were conducted using Student’s *t*-test or Tukey’s test, respectively. All statistical analyses were conducted using IBM SPSS Statistics 26.0 for Windows (IBM Corp., Armonk, NY, USA). Values of *p* < 0.05 were regarded as statistically significant.

## 5. Conclusions

In conclusion, we demonstrated that MPM inhibits cell proliferation by inducing the expression of *let-7c-5p* and *miR-4680-3p* and downregulating their downstream genes. This is the first report to show the involvement of miRNAs in MPM-induced inhibition of palate cells. Since miRNAs are potential therapeutic agents [74], our present investigation may contribute to clinical protocols against CP development. Although further investigation is needed to understand how *let-7c-5p* and *miR-4680-3p* regulate the G1 phase, our findings may aid in understanding the etiology of CP.

## Figures and Tables

**Table 1:** Antibody list for Western blotting.

Antibody Name	Vendor	Catalog Number	Concentration
β-ACTIN	Medical and Biological Laboratories	M177-3	1:3000
BAX	Santa Cruz Biotechnology	sc-20067	1:1000
Cleaved CASPASE-3	Cell Signaling Technology	9661	1:3000
CCND1	Santa Cruz Biotechnology	sc-8396	1:500
CCNE	Santa Cruz Biotechnology	sc-377100	1:1000
CDK2	Santa Cruz Biotechnology	sc-6248	1:1000
CDK4	Santa Cruz Biotechnology	sc-56277	1:1000
CDK6	Santa Cruz Biotechnology	sc-53638	1:500
BACH1	Santa Cruz Biotechnology	sc-271211	1:500
PAX3	Santa Cruz Biotechnology	sc-376204	1:500
ERBB2	Santa Cruz Biotechnology	sc-393712	1:1000
JADE1	Proteintech Japan	28472-1-AP	1:2000
Rabbit IgG HRP	Cell Signaling Technology	7074	1:10,000
Mouse IgG HRP	Cell Signaling Technology	7976	1:10,000

## Data Availability

All relevant data are included within the manuscript.
